# Effects of Tannic Acid Supplementation on Growth Performance, Oocyst Shedding, and Gut Health of in Broilers Infected with *Eimeria Maxima*

**DOI:** 10.3390/ani12111378

**Published:** 2022-05-27

**Authors:** Janghan Choi, Yuguo Huo Tompkins, Po-Yun Teng, Robert M. Gogal, Woo Kyun Kim

**Affiliations:** 1 Department of Poultry Science, University of Georgia, Athens, GA 30602, USA; choij@uga.edu (J.C.); yuguot@uga.edu (Y.H.T.); pyteng@uga.edu (P.-Y.T.); 2 Department of Biosciences and Diagnostic Imaging, College of Veterinary Medicine, University of Georgia, Athens, GA 30602, USA; rgogal@uga.edu

**Keywords:** *Eimeria maxima*, tannins, tannic acid, broilers, gut health, oocyst shedding

## Abstract

**Simple Summary:**

*E. maxima*, an intracellular protozoan parasite propagating in the jejunum, can cause severe negative effects on growth and gut health of chickens. Tannic acid (TA), a polyphenol compound that can precipitate proteins, was hypothesized to control *E. maxima* infection and attenuate its negative effects in broilers due to their antimicrobial, antioxidative, and anti-inflammatory effects. Our study showed that supplementation of TA reduced oocyst shedding of *E. maxima*. Supplementation of TA at the level of 500 to 2750 mg/kg reduced gut permeability, increased nutrient digestibility, and improved intestinal development in broilers infected with *E. maxima*. However, supplementation of 5000 mg/kg TA showed anti-nutritional effects including reducing growth performance, increasing gut permeability, and decreasing nutrient digestibility in broilers infected with *E. maxima*. In summary, supplementation of TA at the level of 500 to 2750 mg/kg showed potential as an anti-coccidial agent by decreasing oocyst shedding and improving nutrient digestibility and intestinal integrity in broilers infected with *E. maxima*.

**Abstract:**

The purpose of this study was to evaluate effects of tannic acid (TA) on growth performance, fecal moisture content, oocyst shedding, gut permeability, lesion score, intestinal morphology, apparent ileal digestibility, and the antioxidant and immune system of broilers infected with *Eimeria maxima*. A total of 420 one-day-old broilers were distributed to five treatments with seven replicates of 12 birds. The five treatments were the (1) sham-challenged control (SCC; birds fed a control diet and administrated with PBS); (2) challenged control (CC; birds fed a control diet and inoculated with *E. maxima*); (3) tannic acid 0.5 (TA0.5; CC + 500 mg/kg TA); (4) tannic acid 2.75 (TA2.75; CC + 2750 mg/kg TA); and (5) tannic acid 5 (TA5; CC + 5000 mg/kg TA). The TA2.75 group had significantly lower gut permeability compared to the CC group at 5 days post-infection (dpi). Supplementation of TA linearly reduced oocyst shedding of *E. maxima* at 7 to 9 dpi (*p* < 0.05). At 13 dpi, the TA2.75 group had significantly greater apparent ileal digestibility (AID) of dry matter (DM) and organic matter (OM) compared to the CC group. At 13 dpi, supplementation of TA linearly increased jejunal villus height (VH). Thus, this study showed that supplementation of TA at levels of 500 to 2750 mg/kg has the potential to be an anti-coccidial agent against *E. maxima* in broilers.

## 1. Introduction

Coccidiosis, induced by the apicomplexan protozoan parasites of the genus *Eimeria*, accounts for more than USD 3 billion in economic loss in the poultry industry annually [1]. The nine identified *Eimeria* spp. in chickens include *E. acervulina*, *E. brunetti*, *E. maxima*, *E. necatrix*, *E. praecox*, *E. mitis*, *E. tenella*, *E. mivati*, and *E. hagani* to date [2,3]. *Eimeria* spp. infect and multiply within the mucosal epithelial layers in the different parts of the gastrointestinal tract of chickens [4]. After several sets of asexual and sexual replications, oocysts are excreted with feces, can be sporulated in the appropriate environment (temperature, humidity, and access to oxygen), and infect chickens repeatedly when they are ingested by chickens [5]. Infection of *E. maxima*, which propagates in the jejunum, can cause severe reduction in nutrient digestion and absorption along with inflammation caused by immune system in broilers [6,7]. Currently, prophylactic coccidiostats and anti-coccidial drugs are provided to control coccidiosis in broilers [8]. However, due to the concern of spread of resistant bacteria and *Eimeria* strains, numerous studies have been conducted to find alternatives for anticoccidial drugs including amino acids [9], prebiotics [10], plant extracts [11], organic acids [12], and nitro compounds [13] in poultry production.

Tannins, polyphenol compounds that can precipitate proteins, are categorized into hydrolysable and condensed tannins. Diverse tannin sources such as chestnut (*Castanea sativa*; hydrolysable tannin) and quebracho (*Schinopsis lorentzii*; condensed tannin) are known to control *Eimeria* infections [14,15,16]. Hydrolysable and condensed tannins have different bioavailability because condensed tannins cannot be hydrolyzed into small molecules in chickens [17]. Although high doses (>5 g/kg) of tannins have cytotoxicity and are considered as anti-nutritional factors in chickens, tannins at appropriate dosages are also known to show beneficial effects by exhibiting strong antimicrobial, antioxidant, and anti-inflammatory effects in chickens [17]. Tannins can limit the growth of microorganisms by directly inhibiting activities of microbial enzymes and by indirectly forming complex with metal ions [18,19]. Moreover, immunomodulatory and antioxidant properties of tannins have potentials to reduce the parasitic infection and attenuate negative impacts of parasitic infections, respectively, in chickens [20,21]. Tannic acid (TA) is considered as the typical and standard of hydrolysable tannins. Tonda et al. [22] reported that supplementation of 500 mg/kg gallnut TA extract reduced total oocysts in excreta and decreased intestinal lesion scores in broilers infected with *E. acervulina*, *E. maxima*, and *E. tenella*. In contrast, Mansoori and Modirsanei [23] showed that supplemental TA (10 g/kg) increased the total number of oocysts in excreta in broilers infected with *Eimeria* spp., which indicates that high dosages of tannins can impair the immune system against coccidiosis and create better gut environment for *Eimeria* propagation. Whether supplementation of TA at appropriate dosages can show antiparasitic effects against *E. maxima* is still unknown. Hence, the purpose of the study was to evaluate effects of different dosages of supplemental TA on growth performance, oocyst shedding, gut permeability, intestinal morphology, number of goblet cells, immune system, and antioxidant capacity in broilers infected with *E. maxima*.

## 2. Materials and Methods

### 2.1. Preparation of E. maxima Inoculum

The inoculum of *E. maxima* was freshly prepared before the inoculation according to the work of Teng et al. [24]. Fresh fecal samples were collected from birds challenged with *E. maxima* in the previous experiments and stored at 4 °C for further steps. To extract *E. maxima* from feces, water was added to the feces and blended thoroughly. Later, feces were removed from blended samples using cheesecloths, and solution was collected. The obtained solution was sedimented overnight and centrifuged at 1000× *g* for 10 min at room temperature to remove supernatant. Afterwards, the saturated salt buffer was added up to 60% of the bottle. The solution was vortexed to suspend all oocysts and then sedimented at room temperature for 30 min. The final samples were centrifuged at 500× *g* for 10 min, and oocysts were collected from the top phase of the supernatant into the new tubes. Water was added to the dilute salt solution and centrifuge at 1000× *g* for 10 min, and the supernatant was discarded. Finally, 2.5% potassium dichromate was added to suspended oocysts, and air was pumped for several days to sporulate oocysts and stored at 4 °C for further steps.

One day before the inoculation, 10^4^ per 1 mL of PBS of *E. maxima* was prepared. To wash 2.5% potassium dichromate, the *E. maxima* solution was centrifuged at 1000× *g* for 10 min, and the supernatant was discarded. The PBS was added and centrifuged at 1000× *g* for 10 min, and the supernatant was discarded. This step was repeated twice to remove potassium dichromate. By using a hemocytometer (Hausser Scientific Company, Horsham, PA, USA), the number of sporulated *E. maxima* was counted, and the appropriate amount of PBS was added to make 10^4^ *E. maxima* per mL.

### 2.2. Experimental Design and Growth Performance

This current study was approved by the Institutional Animal Care and Use Committee at the University of Georgia, Athens, GA, USA. A total of 420 one-day-old Cobb 500 male broilers were randomly allocated to 5 treatments with 7 replicates of 12 birds in each battery cage. As shown in Table 1, diets were formulated to meet or exceed energy and nutrient requirements according to the Cobb Broiler Management Guide (Cobb 2018). The TA was purchased from Sigma-Aldrich (St. Louis, MO, USA) and was added into the filler part with sand to obtain the desired concentrations of TA in the feed. The five experimental treatments were: (1) sham-challenged control (SCC; birds fed a control diet and administrated with PBS); (2) challenged control (CC; birds fed a control diet and inoculated with *E. maxima*); (3) tannic acid 0.5 (TA0.5; CC + 500 mg/kg TA); (4) tannic acid 2.75 (TA2.75; CC + 2750 mg/kg TA); and (5) tannic acid 5 (TA5; CC + 5000 mg/kg TA). Experimental diets were provided during the whole experimental period. Birds had free access to water and feed during the entire experiment period (D 0 to 28), and temperature and light were maintained in accordance with the Cobb Broiler Management Guide (Cobb 2018). Birds and their living conditions were monitored twice daily. For implementing the *E. maxima* challenge model, 10^4^/mL of sporulated *E. maxima* was inoculated via oral gavage to an individual bird on D 15. Body weight (BW) and feed disappearance were recorded to calculate average daily gain (ADG), average daily feed intake (ADFI), and feed conversion ratio (FCR) on D 15, 6 days post-infection (dpi) for the acute phase, and 13 dpi for the recovery phase.

### 2.3. Oocyst Shedding and Fecal Consistency

Fresh fecal samples were collected in sample bags on 3 to 5, 5 to 7, 7 to 9, 9 to 11, and 11 to 13 dpi and stored at 4 °C for further steps. Oocyst shedding was measured according to the work of Teng et al. [25]. Approximately 20 g of feces was weighed and vortexed thoroughly with 20 mL distilled water. One milliliter of samples was mixed with 9 mL of saturated saltwater and vortexed. Finally, the number of *E. maxima* oocysts was counted using a McMaster chamber (Vetlab Supply, Palmetto Bay, FL, USA).

Within 3 to 4 days after sample collection, moisture contents of the ileal and fecal samples were determined as an indicator of fecal consistency according to the work of Koo et al. [26]. Approximately 10 to 15 g of the fecal sample was weighed into the plate and oven-dried at 120 °C for 12 h. Afterwards, the dried samples were weighed. The moisture contents (%) of fecal samples were calculated as follows:

(1) $$\frac{Weight\;of\;the\;fecal\;sample\;before\;drying-Weight\;of\;the\;fecal\;sample\;after\;drying}{Weight\;of\;the\;fecal\;sample\;before\;drying}\times 100\left(\%\right)$$

### 2.4. In Vivo Gut Permeability

At 5 dpi, gut permeability was measured using fluorescein isothiocyanate–dextran (molecular weight: 4 kda; FITC-D4; Sigma-Aldrich Co.) according to the work of Yadav et al. [11]. The 2.2 mg/mL of FITC-D4 was prepared in PBS. One milliliter of the FITC-D4 solution was oral-gavaged to one bird per pen, and after sacrificing birds, blood was collected with heparin-free vacutainers (Grainer Bio-One, Kremsmuenster, Austria) from heart 2 h after FITC-F4 oral gavaging. Collected blood samples were stored in a dark container covered with aluminum foils and kept at room temperature for 1 h to allow clotting. Afterwards, the blood samples were centrifuged at 1000× *g* for 10 min to recover serum. The serum (200 μL) was transferred to a dark 96-well plate, and the fluorescence was measured at an excitation wavelength of 485 nm and emission at 535 nm using a Spectra Max 5 microplate reader (Molecular Devices, Sunnyvale, CA, USA). The concentrations of FITC-D4 were calculated using a prepared standard curve. 

### 2.5. Tissue and Digesta Sample Collection

At 6 dpi, four birds were euthanized by cervical dislocation, and the jejunal lesion from the mid-jejunum to ileum was scored according to the four-score scale described by Johnson and Reid [27]. At 6 and 13 dpi, a 2 cm segment of the mid-jejunum was collected and fixed in a 10% formaldehyde solution. A 10 cm segment of the mid-jejunum was removed and washed with PBS, immediately snap-frozen in liquid nitrogen, and stored at −80 °C for further analyses. Ileal digesta samples were obtained from the upper 10 cm of the ileo-cecal-colic junction to the 10 cm lower Meckel’s diverticulum.

### 2.6. Total Glutathione (GSH) and Oxidized Glutathione (GSSH) Assays, and Total Antioxidant Capacity (TAC) Assay

Total glutathione (GSH) and oxidized glutathione (GSSG) of the mid-jejunal tissues and the liver were measured in duplicate using Caymans GSH assay kits (Cayman Chemical, Ann Arbor, MI, USA). Briefly, 100 mg of mid-jejunal samples was weighted out in a 1.5 mL Eppendorf tube with liquid nitrogen, homogenized in 1 mL of phosphate solution (pH 6.8) containing 1 mM EDTA using a beads beater (Biospec Products, Bartlesville, OK, USA) for 20 s, and then centrifuged at 10,000× *g* for 15 min at 4 °C. Aliquots of the supernatants were taken for the analyses of protein contents using Pierce™ BCA Protein Assay Kits (Thermo Fisher Scientific, Cleveland, OH, USA) after 1:9 dilution. Afterward, metaphosphoric acid (10% *w*/*v*; sigma) was added to the obtained supernatant to precipitate protein, and then the mixture was centrifuged at 3000× *g* for 5 min at room temperature after vortexing. After deproteinization, total GSH and GSSG levels in the resulting supernatant were measured according to the manufacturer’s protocol with following dilutions with sample buffer (jejunal GSH: 1 (sample):9 (buffer); jejunal GSSG 1:1; liver GSH 1:19; and liver GSSG 1:1). Reduced GSH was calculated by the equation: Reduced GSH = Total GSH − 2 × GSSG.

Total antioxidant capacity (TAC) of the mid-jejunal tissues was determined using colorimetric Microplate Assay Kits for Total Antioxidant Capacity (TA02, Oxford Biomedical Research, Oxford, MI, USA) according to the work of Choi et al. [28] with some modifications. Briefly, 100 mg of mid-jejunal samples was weighted out in a 1.5 mL Eppendorf tube with liquid nitrogen, homogenized in 1 mL of PBS (pH 7.0) by a bead beater for 20 s, and then centrifuged at 3000× *g* for 15 min at 4 °C. Aliquots of the supernatants were taken for the analyses of their protein contents using Pierce BCA Protein Assay Kits (Thermo Fisher Scientific) with 1:9 dilution. The TAC analysis was conducted according to the manufacturer’s protocol. The values were expressed as uric acid equivalent mM/mg protein.

### 2.7. Apparent Ileal Digestibility of Nutrients

The concentrations of titanium dioxide in oven-dried samples (0.3 g for the ileal digesta samples and 0.5 g for the feed samples) were analyzed according to the work of Short et al. [29]. The CP content was determined by nitrogen combustion analysis (Method 990.03, AOAC, 2006). Apparent ileal digestibility (AID) of DM, OM, CP, and ash were determined according to the work of Lin and Olukosi [30]. 

### 2.8. Intestinal Morphology and Goblet Cell Counting Analyses

The alcian blue/the period acid-Schiff (AB/PAS) staining was conducted to measure villus height (VH), crypt depth (CD), and the VH/CD ratio, as well as to count the number of goblet cells in VH and CD. Intestinal samples were fixed in 10% neutral-buffered formalin for 72 h and then were stored in the 70% alcohol for further analyses. Afterwards, the jejunal samples were cut. The sections were stained with alcian Blue for 15 min and washed with distilled water. The samples were treated with periodic acid for 5 min and washed with distilled water. Subsequently, the samples were stained with Schiff’s reagent for 10 min and washed with distilled water. Finally, the samples were counterstained in hematoxylin for 1 min and washed and dehydrated. The stained sections were pictured with a microscope (Leica DC500 camera, Leica Microsystems Inc., Buffalo Groove, IL, USA). Images (4×) were analyzed using ImageJ (National Institutes of Health, Bethesda, MD, USA). 

### 2.9. RNA Extraction and Real-Time Polymerase Chain Reaction (PCR) Analysis

Approximately 100 mg of the whole jejunum samples were homogenized in QIAzol lysis reagents (Qiagen, Valencia, CA, USA), and RNAs were extracted according to the manufacturer’s procedure. A NanoDrop 2000 spectrophotometer (Thermo Fisher Scientific) was used to determine RNA quantity and purity. One microgram of RNA was used to produce the first-strand cDNA using high-capacity cDNA synthesis kits (Applied Biosystems, Foster City, CA, USA). Primers used in the study are shown in Table 2. Real-time PCR was performed using SYBR Green Master Mix (Invitrogen, Carlsbad, CA, USA) with a Step One thermocycler (Applied Biosystem). The final PCR volume (10 μL) contained 5 μL of SYBR Green Master Mix, 1.5 μL of cDNA, 0.5 μL of forward and reverse primers (10 μM), and 2.5 μL of water. The thermal cycle condition for all reactions was as follows: 95 °C denature for 10 min, 40 cycles at 95 °C for 15 s and 60 °C for 1 min, 95 °C for 15 s, 60 °C for 1 min and 95 °C for 15 s. Several PCR products from each were electrophoresed on a 3% agarose gel in Tris-acetate-EDTA buffer and visualized by adding SYBR green (Invitrogen), and a melting curve of each gene was checked to confirm the specificity of each PCR product. The glyceraldehyde 3-phosphate dehydrogenase (GAPDH) and beta actin were used as the housekeeping genes (reference genes). The target mRNA abundance was normalized with geometric means of housekeeping genes [31]. Relative mRNA abundance was determined by using the 2^−∆∆Ct^ method [32]. The negative control, containing no cDNA, was included in each run, and each sample was run in duplicate. 

### 2.10. Statistical Analyses

Statistical analyses were conducted utilizing SAS (version 9.4; SAS Inst. Inc., Cary, NC, USA). Data normality was checked using proc univariate except for lesion score data. The SCC and CC groups were compared using Student’s t-test. *E. maxima*-infected groups were compared using one-way ANOVA in a completely randomized design followed by Tukey’s comparison test. Orthogonal polynomial contrasts analysis was conducted to see linear pattern (L) and quadratic pattern (Q) among *Eimeria maxima* challenged groups. The Kruskal–Wallis test followed by the Dwass–Steel–Critchlow–Fligner post hoc test was performed to analyze lesion score data. Significance level was set at *p* < 0.05, and tendencies were also presented at 0.05 < *p* ≤ 0.10 [33]. 

## 3. Results

### 3.1. Growth Performance

As shown in Table 3, the TA5 birds had the reduced BW and ADG compared to the CC and TA0.5 groups (*p* < 0.05), and BW, ADG, and ADFI were linearly reduced by supplementation of TA in the pre-challenge period (*p* < 0.05). In the acute phase (0 to 6 dpi), *E. maxima* infection significantly decreased BW, ADG and ADFI in broilers. The TA0.5 group had the significantly higher BW compared to the TA5 group, and BW was linearly reduced as TA supplementation dosage increased (*p* < 0.01). In the recovery phase (6 to 13 dpi), the CC group had statistically similar BW compared to the SCC group (*p* > 0.1) but had significantly higher ADFI compared to the SCC broilers. The TA5 group had the reduced BW at 13 dpi compared to the CC and TA0.5 groups (*p* < 0.05), and supplementation of TA linearly reduced BW, ADG, and FCR in broilers infected with *E. maxima* (*p* < 0.01).

### 3.2. Fecal Moisture Content and Oocyst Shedding

Inoculation of *E. maxima* did not affect the ileal and fecal moisture contents at all time points, as shown in Table 4 (*p* > 0.1). At 5 to 7 dpi, the TA2.75 group had a decreased fecal moisture content compared to the CC group, and supplementation of TA linearly and quadratically reduced fecal moisture content (*p* < 0.05). At 9 to 11 dpi and 11 to 13 dpi, the TA5 group had decreased fecal moisture content compared to the TA0.5 group, and supplementation of TA linearly reduced fecal moisture content (*p* < 0.01).

**Figure 1:**
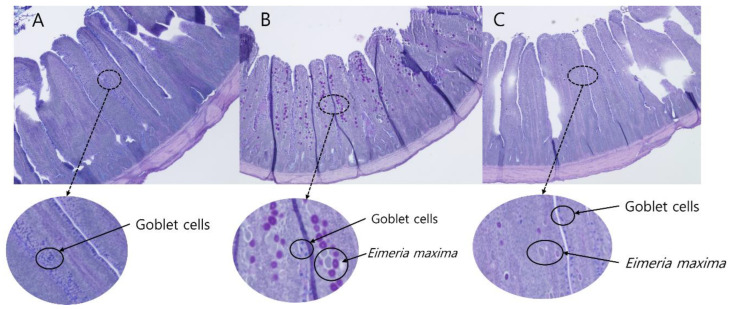
Alcian blue/the period acid-Schiff (AB/PAS)-stained jejunal morphology of (**A**) SCC (sham-challenged control): chickens fed a control diet and challenged with phosphate-buffered saline; (**B**) CC (challenged control): chickens fed a control diet and challenged with 10^4^ of *Eimeria maxima*; and (**C**) TA0.5 (tannic acid 500 mg/kg): CC + 500 mg/kg tannic acid. Small purple dots are goblet cells, and big white and purple dots are *E. maxima*. The SCC group had no *E. maxima* cross-contamination, and the TA0.5 group had less *E. maxima* in the mucus layer compared to the CC group.

As shown in Table 5, in the SCC group, *E. maxima* oocysts were not detected in feces at all time points. At 5 to 7 dpi, supplementation of TA tended to linearly reduce oocyst shedding in broilers (*p* = 0.09); much less *E. maxima* in the jejunal section of the 0.5TA group was observed compared to the CC group (Figure 1). At 7 to 9 dpi, TA0.5, TA2.75, and TA5 groups had significantly lower oocyst shedding compared to the CC group. However, differences in oocyst shedding were not observed at 9 to 11 dpi among the treatments (*p* > 0.1). 

### 3.3. Gut Permeability and Jejunal Lesion

The inoculation of *E. maxima* increased the serum FITC-D4 concentration in broilers (*p* < 0.05), which indicates higher gut leakage (Table 6). The TA2.75 group had significantly reduced serum FITC-D4 concentrations than the CC group, and supplementation of TA quadratically reduced serum FITC-D4 concentrations (*p* < 0.05). As shown in Table 6, *E. maxima* inoculation induced mild jejunal lesion in broilers (*p* < 0.05). However, supplementation of TA did not modulate jejunal lesion in broilers infected with *E. maxima* (*p* > 0.1).

### 3.4. Total Antioxidant Capacity (TAC), Total Glutathione (GSH), Reduced GSH, and Oxidized Glutathione (GSSG)

*E. maxima* infection did not affect TAC of the jejunum at 6 dpi (*p* > 0.1; Table 7). Supplementation of TA tended to linearly reduce TAC of the jejunum (*p* = 0.08). At 6 dpi, supplementation of TA linearly decreased total GSH (*p* < 0.05) and reduced GSH (tendency; *p* = 0.07) and increased GSSG (tendency; *p* = 0.05) in the jejunum of broilers infected with *E. maxima*. At 6 dpi, *E. maxima* infection tended to linearly decrease reduced GSH (*p* = 0.09) and significantly decreased reduced GSH/GSSG in the liver (*p* < 0.05). At 13 dpi, the TA2.75 group had lower total GSH and reduced GSH in the jejunum compared to the TA0.5 group (*p* < 0.05), and the supplementation of TA tended to linearly decrease total GSH (*p* = 0.07) and reduced GSH (*p* = 0.1) and linearly increase GSSG (*p* = 0.1) in the jejunum. At 13 dpi, *E. maxima* infection tended to increase GSSG (*p* = 0.06) and decreased reduced GSH/GSSG (*p* = 0.06) in the liver. The reduced GSH in the liver tended to be linearly decreased due to supplementation of TA (*p* = 0.1).

### 3.5. Apparent Ileal Digestibility of Nutrients

As shown in Table 8, *E. maxima* infection significantly increased AID of ash, and the TA0.5 and TA5 groups had lower AID of ash compared to the CC group at 6 dpi. At 6 dpi, the TA5 group had lower AID of ash compared to the TA2.75 group. Supplementation of TA linearly decreased AID of ash at 6 dpi (*p* < 0.05). However, no differences were observed in AID of DM and OM among the treatments at 6 dpi (*p* > 0.1). At 13 dpi, the CC broilers had significantly greater AID of DM, OM, and ash compared to the SCC broilers (*p* < 0.05). The TA2.75 group had significantly higher AID of DM and OM compared to the CC group. However, the TA5 group had the lowest AID of DM and OM among the *E. maxima*-infected broilers. The TA0.5 and TA5 groups had significantly lower AID of ash compared to the CC and TA2.75 groups. No differences were observed in AID of CP at 13 dpi among the treatments (*p* > 0.1). Supplementation of TA linearly decreased AID of DM, OM, and ash in broilers infected with *E. maxima* (*p* < 0.05).

### 3.6. Jejunal Morphology and Goblet Cell Density

*E. maxima* infection significantly reduced jejunal VH and the VH/CD ratio when CC birds were compared to the SCC birds at 6 dpi (Table 9, Figure 1). At 13 dpi, jejunal VH and VH/CD ratio were decreased by *E. maxima* infection (*p* < 0.05), whereas jejunal CD and goblet cells per 100 μm VH and per 100 μm CD of the CC group were increased compared to the SCC group (*p* < 0.05). Supplementation of TA linearly increased jejunal VH in broilers infected with *E. maxima* (*p* < 0.05).

### 3.7. Relative mRNA Expression

**Figure 2:**
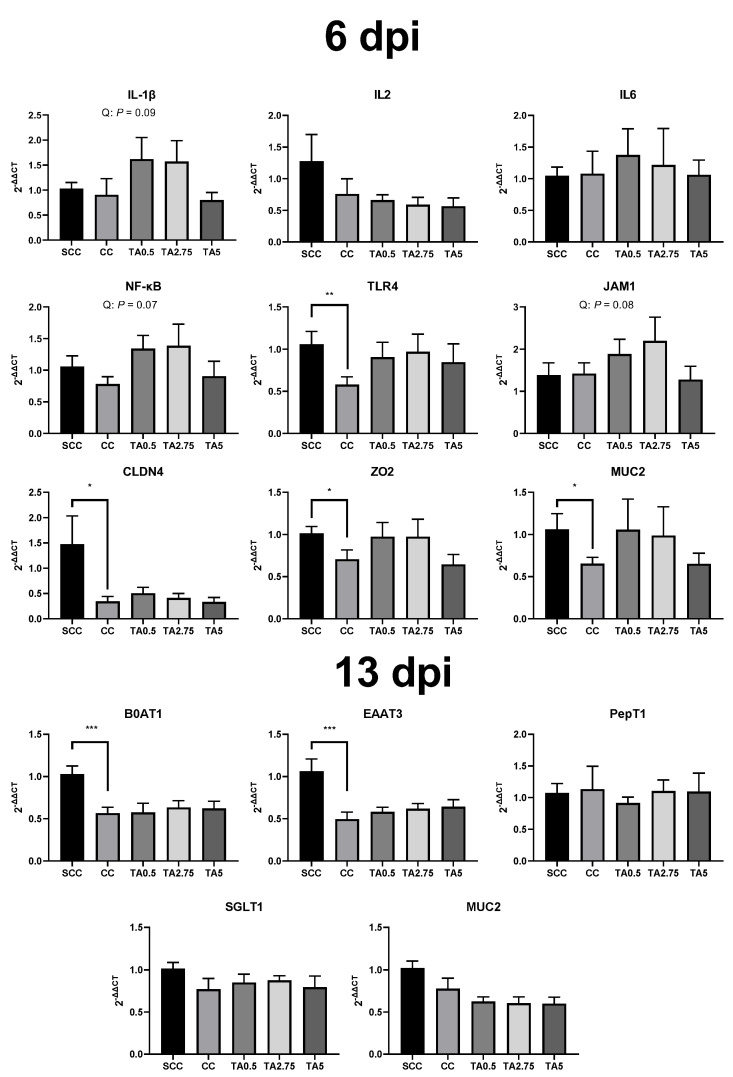
Relative mRNA gene expression in the jejunum at 6 days post-infection (dpi) (genes related to immune system, gut barrier integrity, and mucin) and 13 dpi (nutrient transporter and mucin) in the SCC ((sham-challenged control): broilers fed a control diet and administrated with phosphate buffered saline via oral gavage); CC ((challenged control): broilers fed a control diet and administrated with 10^4^ of *Eimeria maxima* via oral gavage); TA0.5 ((tannic acid 500 mg/kg): CC + 500 mg/kg of tannic acid); TA2.75 ((tannic acid 2750 mg/kg): CC + 2750 mg/kg of tannic acid); TA5 ((tannic acid 5000 mg/kg): CC + 5000 mg/kg of tannic acid) groups. Each value represents the mean ± SEM. SCC was compared with CC (unpaired *t*-test), and the comparison was presented as * 0.05 < *p* < 0.10, ** *p* < 0.05, *** *p* < 0.01. Bars with different letters are significantly different (*p* < 0.05) by PROC MIXED followed by the Tukey’s multiple comparison test among *E. maxima*-infected groups (CC, TA0.5, TA2.75, and TA5). Orthogonal polynomial contrasts analysis was conducted to see linear pattern (L) and quadratic pattern (Q) among *Eimeria maxima* challenged groups. GAPDH, glyceraldehyde 3-phosphate dehydrogenase; NFκB, nuclear factor kappa-light-chain-enhancer of activated B cells; IL, interleukin; JAM2, junctional adhesion molecule 2; ZO2, zonula occludens 2; CLDN4, claudin 4; SGLT1, sodium glucose transporter 1; PepT1, peptide transporter 1; B0AT1, sodium-dependent neutral amino acid transporter 1; EAAT3, excitatory amino acid transporter 3; GLUT2, glucose transporter 2; and MUC2, mucin 2.

Due to *E. maxima* infection, relative mRNA expression of toll-like receptor 4 (TLR4) was significantly reduced, and relative mRNA expression of zonula occludens 2 (ZO2), claudins 4 (CLDN4), and mucin 2 (MUC2) of the CC group tended to be decreased (*p* = 0.05, *p* = 0.07, and *p* = 0.05, respectively; Figure 2) at 6 dpi. Supplementation of TA tended to quadratically increase relative mRNA expression of interleukin 1β (IL1β; *p* = 0.09), nuclear factor kappa-light-chain-enhancer of activated B cells (NFκB; *p* = 0.07), and junctional adhesion molecule 2 (JAM2; *p* = 0.08) at 6 dpi. During the recovery phase, the CC broilers had significantly lower relative mRNA expression of sodium-dependent neutral amino acid transporter (B0AT1) and excitatory amino acid transporter 3 (EAAT3) compared to the SCC broilers. Nevertheless, no differences were observed among the *E. maxima* infected groups.

## 4. Discussion

The purpose of the current study was to investigate effects of TA on growth performance, intestinal morphology, goblet cell number, gut barrier integrity, antioxidant capacity, oocyst shedding, and immune system in broilers infected with *E. maxima. E. maxima* infection significantly decreased BW, ADG, and ADFI in the acute phase, which is consistent with our previous study [24]. During the recovery phase, the CC group had statistically similar BW to the SCC group by enhancing feed intake and nutrient digestibility in the current study, which implies that there was compensatory growth in the CC group. Supplementation of TA reduced BW and ADG during the pre-challenge period via decreasing the feed intake. The TA–saliva protein complexes are known to induce astringent and bitter taste, which may decrease feed intake of broilers [34]. Moreover, compromised gut ecosystem due to the high concentrations of TA potentially reduces feed intake of broilers [35]. During the acute phase, supplementation of TA linearly decreased BW of broilers without affecting feed intake, intestinal morphology, and nutrient digestibility of broilers, which suggests that reduced growth performance by supplementation of TA at young ages may continuously affect growth performance of broilers at later growing phases. However, the TA0.5 group had the numerically highest BW among the *E. maxima*-infected groups, which suggests that supplementation of 500 mg/kg TA may have potential to improve growth performance of broilers infected with *E. maxima*. In contrast, supplementation of TA linearly reduced BW, ADG, and FCR of broilers infected with *E. maxima* in the recovery phase. In the current study, supplementation of TA did not reduce ADG, ADFI, and FCR in the acute phase potentially because TA exhibited defensive effects against *E. maxima* infection in broilers. 

Supplementation of TA decreased oocyst shedding of *E. maxima* in broilers at 5 to 9 dpi. Consistent with a previous study [36], around 7 dpi was the peak point for oocyst shedding of *E. maxima.* Moreover, pictures of jejunum morphology showed visually less *E. maxima* in the TA0.5 group compared to the CC group (Figure 1). In agreement with this, Tonda et al. [22] and Kaleem et al. [37] reported that tannin extract from plants decreased oocyst shedding in broilers infected with *Eimeria* spp. Because there were no differences in the jejunal lesion score among the groups infected with *E. maxima*, thereby, TA could potentially not inhibit the invasion of *E. maxima* into the enterocytes but instead inhibit the sexual reproduction of *E. maxima* to produce oocysts. Sexual reproduction of *Eimeria* spp., following at least two cycles of asexual reproductions, is required to produce oocysts [38]. Potentially, TA may directly inhibit enzymes activities and interact with proteins related to sexual reproduction or deprive nutrients (e.g., iron and proteins) from parasites for their reproduction by forming complex with proteins and metals. Reducing oocyst shedding is an important trait as an anti-coccidial agent because this possibly reduces continuous exposure of *E. maxima* in a chicken flock [36].

We hypothesized that *E. maxima* infection may increase ileal and fecal moisture content because *E. maxima* is known to induce diarrhea in broilers [39]. However, the inoculation dosage used in the current study induced only mild infection and did not lead to watery digesta and feces. Because *E. maxima* infection did not induce diarrhea in the present study, decreased ileal and fecal moisture content might not have been due to anti-diarrhea effects of TA. Reduced moisture content in ileal digesta (9 dpi) and feces (9 to 13 dpi) could be closely associated with drastically reduced AID of ash in the current study. Tannins form a complex with minerals (zinc, iron, copper, etc.), which potentially results in decreased mineral utilization and increased endogenous loss of minerals in chickens [40]. Secretion and absorption of electrolytes are closely related to water secretion and absorption in the intestine, and ileal and fecal moisture contents were reduced with the similar trends with the AID of ash in the current study. Reducing digesta and fecal moisture content via reducing AID of ash are not considered beneficial effects because loss of ash can result in reduced growth performance and bone development in broilers. 

Gut permeability, measured by the FITC-D4 gavage method, was increased by *E. maxima* infection, consistent with our previous study [11]. Increased gut permeability, which is mainly modulated by tight junction proteins (ZO2, CLDN4, JAM2, etc.) and mucus (main protein in the intestine, MUC2) [41], indicates that pathogenic bacteria and toxins are more likely to enter the bloodstream of broilers. [42]. Consistent with the FITC-D4 permeability assay result, *E. maxima* infection reduced mRNA expression of ZO2, CLDN4, and MUC2 in the present study. Teng et al. [24] reported that during the *E. maxima* asexual and sexual replication, tight junction proteins between enterocytes can be damaged. Supplementation of TA quadratically decreased gut permeability in broilers infected with *E. maxima*, and the TA2.75 group had significantly lower gut permeability compared to the CC group in the present study. In agreement with this, supplementation of TA tended to quadratically increase relative mRNA expression of ZO2 in the current study. Along with increased mRNA expression of tight junction proteins, supplementation of TA quadratically increased relative mRNA expression of IL1β and NFκB. Increased mRNA expression of IL1β and NFκB levels have been considered as negative factors for intestinal barrier integrity [43,44]. However, potentially pro-inflammatory cytokines are still important in controlling several pathogens by activating the immune system and maintaining gut barrier integrity as defensive mechanisms in broilers infected with *Eimeria* spp. [45]. However, broilers fed 5000 mg/kg TA (TA5) increased gut permeability compared to the broilers fed 2750 mg/kg TA (TA2.75) in the present study. This may have been because high concentrations of TA can show cytotoxicity and impair tight junction proteins. These results indicate that supplementation of TA at appropriate dosages improves gut barrier integrity via modulating mRNA expression of tight junction proteins and stimulating the immune system of broilers infected with *E. maxima*.

In the recovery phase, AID of DM, OM, and ash were significantly increased in the CC group compared to the SCC group, which supports the fact that there was a compensatory growth in the recovery phase after mild infection of *E. maxima* in the study. The AID of DM and CP were linearly reduced and quadratically increased due to supplementation of TA in the present study. It is well known that TA decreases nutrient digestibility by compromising gut health and forming a complex with proteins in monogastric animals [46]. However, the TA2.75 group had the higher AID of DM and OM compared to the CC group, indicating that TA at appropriate dosages has the potential to increase nutrient digestibility in broilers, depending on the conditions. 

The GSH is the major endogenous antioxidant in the cells of broilers [47]. A decrease in total GSH, reduced GSH, and GSH/GSSG and an increase in GSSG in the jejunum and liver by supplementation of TA in the current study indicates that TA can impair the endogenous antioxidant system and induce oxidative stress in broilers infected with *E. maxima*. However, the TA0.5 group had numerically similar or even better values in GSH, GSSG, reduced GSH, and reduced GSH/GSSG compared to the CC group. Many studies showed that tannins have antioxidant capacity in animals [48,49]. However, our current study showed that cytotoxic and proteolytic effects [50] of TA at high concentrations can induce oxidative stress and impair the endogenous antioxidant system in broilers infected with *E. maxima*.

In the current study, *E. maxima* infection reduced jejunal morphology at 6 and 13 dpi in broilers, indicating restricted nutrient absorption and digestion, which is in agreement with our previous study [24]. Supplementation of TA quadratically increased CD of broilers infected with *E. maxima* at 6 dpi; however, this did not lead to changes in the VH and VH/CD ratio in the current study. At 13 dpi, supplementation of TA linearly increased jejunal VH in the current study, which potentially explains the increased nutrient digestibility. Wang et al. [51] reported that supplementation of TA increased jejunal development in mice challenged with diquat (an oxidative stress model). The differences were due to different animals, challenge models (parasitic infection vs. oxidative stress model), and ways to provide TA (feeding vs. oral gavage). Broilers infected with *E. maxima* had increased goblet cell density in the present study. Goblet cells, which produce mucus, play important roles in cytoprotective functions against colonization of pathogens in the epithelium of the small intestine [26]. However, over-produced mucus can increase bacterial pathogenesis because some pathogens can use mucus as their food and habitat [52,53]. Collier et al. [54] reported that *E. maxima* caused a host mucogenic response, which can make the intestine vulnerable to be infected with pathogens (e.g., *Clostridium perfringens*) that utilize mucus for their growth and proliferation in the intestine of broilers. However, supplementation of TA did not modulate the concentrations of goblet cells in the villus and crypts in the present study. 

## 5. Conclusions

*E. maxima* infection significantly decreased growth performance and impaired gut ecosystem of broilers. Whereas supplementation of TA at high concentrations (5000 mg/kg) resulted in decreased growth performance, nutrient digestibility, antioxidant system, and gut barrier integrity, 500 to 2750 mg/kg of TA resulted in reduced oocyst shedding, activated immune system, enhanced gut barrier integrity, and improved intestinal morphology and nutrient digestibility in broilers infected with *E. maxima*. Therefore, supplementation of TA at levels of 500 to 2750 mg/kg has the potential to be an anti-coccidial agent and improve gut health in broilers.

## Figures and Tables

**Table 1:** Ingredients and nutrient compositions of basal diets (as-fed basis).

Items	D 0 to 15	D 15 to 28
Ingredients (kg/ton)		
Corn	651.95	700.80
Soybean meal (480 g crude protein /kg)	294.94	241.76
Defluorinated phosphate	15.78	15.84
Filler ^1^ (sand and tannic acid)	10.00	13.99
Soybean oil	7.93	10.00
Limestone	7.16	6.11
DL-Methionine 99%	3.17	2.85
L-Lysine HCl 78%	3.01	2.80
Vitamin premix ^2^	2.50	2.50
Common salt	1.55	1.79
L-Threonine	1.20	0.80
Mineral premix ^3^	0.80	0.77
Total	1000	1000
Calculated energy and nutrient value, %		
Metabolizable energy, Mcal/kg	3000	3100
Crude protein, %	20.60	18.37
SID ^4^ methionine, %	0.61	0.55
SID ^4^ total sulfur amino acids, %	0.88	0.80
SID ^4^ lysine, %	1.17	1.02
SID ^4^ threonine, %	0.78	0.66
Total calcium, %	0.87	0.76
Available phosphorus, %	0.44	0.38

^1^ Sand and tannic acid were added to obtain desired tannic acid dosage in the feed as follows (control: sand 10 g/kg + tannic acid 0 g/kg, TA0.5: sand 9.5 g/kg + tannic acid 0.5 g/kg, TA2.75: sand 7.25 g/kg + tannic acid 2.75 g/kg, and TA5: sand 5 g/kg + tannic acid 5 g/kg). ^2^ Vitamin mix provided the following in mg/100 g diet: thiamine-HCl, 1.5; riboflavin 1.5; nicotinic acid amide 15; folic acid 7.5; pyridoxine-HCl, 1.2; d-biotin 3; vitamin B-12 (source concentration, 0.1%), 2; d-calcium pantothenate, 4; menadione sodium bisulfite, 1.98; α-tocopherol acetate (source 500,000 IU/g), 22.8; cholecalciferol (source 5000,000 IU/g), 0.09; retinyl palmitate (source 500,000 IU/g), 2.8; ethoxyquin, 13.34; I-inositol, 2.5; dextrose, 762.2. ^3^ Mineral mix provided the following in g/100 g diet: Ca(H_2_PO_4_)_2_·H_2_O, 3.62; CaCO_3_, 1.48; KH_2_PO_4_, 1.00; Na_2_SeO_4_, 0.0002; MnSO_4_·H_2_O, 0.035; FeSO_4_·7H_2_O, 0.05; MgSO_4_·7H_2_O, 0.62; KIO_3_, 0.001; NaCl, 0.60; CuSO_4_·5H_2_O, 0.008; ZnCO_3_, 0.015; CoCl_2_·6H_2_O, 0.00032; NaMoO_4_·2H_2_O, 0.0011; KCl, 0.10; dextrose, 0.40. ^4^ SID: standard ileal digestible amino acid.

**Table 2:** Primers used in the study ^1^.

Genes	Sequence, 5′ to 3′	Amplicon
GAPDH	F: GCT AAG GCT GTG GGG AAA GT	161
	R: TCA GCA GCA GCC TTC ACT AC	
Beta actin	F: CAA CAC AGT GCT GTC TGG TGG TA	205
	R: ATC GTA CTC CTG CTT GCT GAT CC	
NFκB	F: GAA GGA ATC GTA CCG GGA ACA	131
	R: CTC AGA GGG CCT TGT GAC AGT AA	
IL1β	F: TGC CTG CAG AAG AAG CCT CG	204
	R: GAC GGG CTC AAA AAC CTC CT	
IL2	F: TTG GCT GTA TTT CGG TAG CA	169
	R: GTG CAC TCC TGG GTC TCA GT	
IL6	F: ATA AAT CCC GAT GAA GTG G	146
	R: CTC ACG GTC TTC TCC ATA AA	
JAM2	F: AGC CTC AAA TGG GAT TGG ATT	59
	R: CAT CAA CTT GCA TTC GCT TCA	
ZO2	F: ATC CAA GAA GGC ACC TCA GC	100
	R: CAT CCT CCC GAA CAA TGC	
CLDN4	F: GAA GCG CTG AAA CGA TAC CA	134
	R: TGC TTC TGT GCC TCA GTT TCC	
SGLT1	F: GCC ATG GCC AGG GCT TA	71
	R: CAA TAA CCT GAT CTG TGC ACC AGT A	
PEPT1	F: CCC CTG AGG AGG ATC CTT	66
	R: CAA AAG AGC AGC AAC GA	
B0AT1	F: GGG TTT TGT GTT GGC TTA GGA A	60
	R: TCC ATG GCT CTG GCA GAG AT	
EAAT3	F: TGC TGC TTT GGA TTC CAG TGT	79
	R: AGC AAT GAC TGT AGT GCA GAA GTA ATA TAT G	
GLUT2	F: TCA TTG TAG CTG AGC TGT T	147
	R: CGA AGA CAA CGA ACA CAT AC	
MUC2	F: ATG CGA TGT TAA CAC AGG ACT C	110
	R: GTG GAG CAC AGC AGA CTT TG	

^1^ GAPDH, glyceraldehyde 3-phosphate dehydrogenase; NFκB, nuclear factor kappa-light-chain-enhancer of activated B cells; IL, interleukin; JAM2, junctional adhesion molecule 2; ZO2, zonula occludens 2; CLDN4, claudin 4; SGLT1, sodium glucose transporter 1; PepT1, peptide transporter 1; B0AT1, sodium-dependent neutral amino acid transporter 1; EAAT3, excitatory amino acid transporter 3; GLUT2, glucose transporter 2; MUC2, mucin 2.

**Table 3:** Effects of tannic acid supplementation on growth performance parameters including body weight (BW, g), average daily gain (ADG, g/d), average daily feed intake (ADFI, g/d), and feed conversion ratio (FCR, g/g) of broilers infected with *Eimeria maxima* during the pre-challenge period (D 0 to 15), acute phase: 0 to 6 days post-infection (dpi), and recovery phase: 6 to 13 dpi ^1^.

		*Eimeria maxima*-Challenged ^3^						Poly. Contrast	
Items	SCC ^2^	CC	TA0.5	TA2.75	TA5	SEM	*p*-Value	Lin.	Quad.
Pre-challenge									
BW	462.1	440.8 ^a^	452.8 ^a^	432.8 ^ab^	404.7 ^b^	19.9	<0.01	<0.01	0.19
ADG	27.68	26.26 ^a^	27.06 ^a^	25.7 ^ab^	23.8 ^b^	1.4	<0.01	<0.01	0.22
ADFI	38.96	38.37	38.46	37.11	35.85	2.02	0.07	0.01	0.88
FCR	1.41	1.46	1.42	1.44	1.51	0.11	0.52	0.25	0.41
Acute phase									
BW	812.2 ***	696.5 ^ab^	721.4 ^a^	691.9 ^ab^	638.5 ^b^	41.7	<0.01	<0.01	0.15
ADG	58.3 ***	42.6	44.7	43.2	39	5.5	0.28	0.11	0.31
ADFI	115.4 ***	86.8	87.5	92.3	87.9	7.51	0.52	0.59	0.18
FCR	1.99	2.08	1.96	2.18	2.33	0.48	0.80	0.36	0.90
Recovery phase									
BW	1273	1211 ^a^	1227 ^a^	1141 ^ab^	1062 ^b^	105.3	<0.01	<0.01	0.41
ADG	69.8	75.6	74.2	64.3	57.6	23.5	0.04	<0.01	0.88
ADFI	118 **	130.2	128.1	124	121	11.7	0.47	0.12	0.82
FCR	1.75	1.75	1.76	1.94	2.2	0.32	0.04	<0.01	0.72

^1^ SCC (sham-challenged control): broilers fed a control diet and administrated with phosphate-buffered saline via oral gavage; CC (challenged control): broilers fed a control diet and administrated with 10^4^ of *Eimeria maxima* via oral gavage; TA0.5 (tannic acid 500 mg/kg): CC + 500 mg/kg of tannic acid; TA2.75 (tannic acid 2750 mg/kg): CC + 2750 mg/kg of tannic acid; and TA5 (tannic acid 5000 mg/kg): CC + 5000 mg/kg of tannic acid. ^2^ SCC vs. CC (unpaired t-test): ** *p* < 0.05, *** *p* < 0.01. ^3^ Different letters are significant different (*p* < 0.05) by PROC mixed followed by the Tukey’s multiple comparison test among *Eimeria maxima* challenged groups (CC, TA0.5, TA2.75, and TA5). Orthogonal polynomial contrasts analysis was conducted to see linear pattern (L) and quadratic pattern (Q) among *Eimeria maxima* challenged groups.

**Table 4:** Effects of tannic acid supplementation on ileal and fecal moisture content (%) of broilers infected with *Eimeria maxima* at 6 and 13 days post-infection (dpi) for ileal samples and 3 to 5 dpi, 5 to 7 dpi, 7 to 9 dpi, 9 to 11 dpi, and 11 to 13 dpi for fecal samples ^1^.

		*Eimeria maxima*-Challenged ^3^						Poly. Contrast	
Items	SCC ^2^	CC	TA0.5	TA2.75	TA5	SEM	*p*-Value	Lin.	Quad.
Ileal content									
6 dpi	81.64	82.46	84.24	82.02	82.69	2.03	0.22	0.44	0.63
13 dpi	81.46	81.99	82.00	81.23	80.45	1.2	0.07	0.01	0.86
Fecal content									
3 to 5 dpi	75.47	75.30	78.23	75.68	76.09	4.44	0.62	0.77	0.96
5 to 7 dpi	78.84	76.77 ^a^	74.84 ^a^	68.69 ^b^	72.41 ^ab^	3.92	<0.01	0.01	<0.01
7 to 9 dpi	78.85	78.00	79.65	76.00	77.28	2.54	0.09	0.13	0.22
9 to 11 dpi	80.04	80.06 ^ab^	82.11 ^a^	79.74 ^ab^	77.26 ^b^	2.54	0.01	<0.01	0.26
11 to 13 dpi	77.77	79.32 ^a^	79.79 ^a^	77.24 ^ab^	74.80 ^b^	2.70	<0.01	<0.01	0.72

^1^ SCC (sham-challenged control): broilers fed a control diet and administrated with phosphate-buffered saline via oral gavage; CC (challenged control): broilers fed a control diet and administrated with 10^4^ of *Eimeria maxima* via oral gavage; TA0.5 (tannic acid 500 mg/kg): CC + 500 mg/kg of tannic acid; TA2.75 (tannic acid 2750 mg/kg): CC + 2750 mg/kg of tannic acid; and TA5 (tannic acid 5000 mg/kg): CC + 5000 mg/kg of tannic acid. ^2^ SCC and CC were compared by using unpaired *t*-test. ^3^ Different letters are significant different (*p* < 0.05) by PROC mixed followed by the Tukey’s multiple comparison test among *Eimeria maxima* challenged groups (CC, TA0.5, TA2.75, and TA5). Orthogonal polynomial contrasts analysis was conducted to see linear pattern (L) and quadratic pattern (Q) among *Eimeria maxima* challenged groups.

**Table 5:** Effects of tannic acid supplementation on oocyst shedding (number of oocysts per gram in feces) in broilers infected with *Eimeria maxima* at 5 to 7 days post-infection (dpi), 7 to 9 dpi, and 9 to 11 dpi ^1^.

		*Eimeria maxima*-Challenged ^2^						Poly. Contrast	
Items	SCC	CC	TA0.5	TA2.75	TA5	SEM	*p*-Value	Lin.	Quad.
5 to 7 dpi	N/D ^3^	2205.6	2198.6	371.3	426.1	2446.8	0.31	0.09	0.47
7 to 9 dpi		6203.8 ^a^	686.3 ^b^	680.8 ^b^	452.1 ^b^	3719.2	0.02	0.04	0.14
9 to 11 dpi		827.2	1143.6	272.9	963.8	1140.7	0.52	0.78	0.26

^1^ SCC (sham-challenged control): broilers fed a control diet and administrated with phosphate-buffered saline via oral gavage; CC (challenged control): broilers fed a control diet and administrated with 10^4^ of *Eimeria maxima* via oral gavage; TA0.5 (tannic acid 500 mg/kg): CC + 500 mg/kg of tannic acid; TA2.75 (tannic acid 2750 mg/kg): CC + 2750 mg/kg of tannic acid; and TA5 (tannic acid 5000 mg/kg): CC + 5000 mg/kg of tannic acid.^2^ Different letters are significant different (*p* < 0.05) by PROC mixed followed by the Tukey’s multiple comparison test among *Eimeria maxima* challenged groups (CC, TA0.5, TA2.75, and TA5). Orthogonal polynomial contrasts analysis was conducted to see linear pattern (L) and quadratic pattern (Q) among *Eimeria maxima* challenged groups. ^3^ N/D: not detected.

**Table 6:** Effects of tannic acid supplementation on serum FITC D4 concentration and *Eimeria maxima* lesion in broiler chickens infected with *Eimeria maxima* at 5 days post-infection (dpi) and 6 dpi, respectively ^1^.

		*Eimeria maxima*-Challenged ^3^						Poly. Contrasts	
Items	SCC ^2^	CC	TA0.5	TA2.75	TA5	SEM	*p*-Value	Lin.	Quad.
Serum FITC-D4 ^3^	0.15 ***	2.22 ^a^	1.76 ^ab^	1.06 ^b^	2.04 ^a^	0.10	<0.01	0.46	<0.01
Jejunal lesion ^4^	0 ***	0.75	0.96	0.78	0.82	0.02	0.62	0.84	0.96

^1^ SCC (sham-challenged control): broilers fed a control diet and administrated with phosphate-buffered saline via oral gavage; CC (challenged control): broilers fed a control diet and administrated with 10^4^ of *Eimeria maxima* via oral gavage; TA0.5 (tannic acid 500 mg/kg): CC + 500 mg/kg of tannic acid; TA2.75 (tannic acid 2750 mg/kg): CC + 2750 mg/kg of tannic acid; TA5 (tannic acid 5000 mg/kg): CC + 5000 mg/kg of tannic acid. ^2^ SCC vs. CC (unpaired *t*-test): *** *p* < 0.01. ^3^ Different letters are significant different (*p* < 0.05) by PROC mixed followed by the Tukey’s multiple comparison test among *Eimeria maxima* challenged groups (CC, TA0.5, TA2.75, and TA5). Orthogonal polynomial contrasts analysis was conducted to see linear pattern (L) and quadratic pattern (Q) among *Eimeria maxima* challenged groups.^4^ The Kruskal–Wallis test followed by the Dwass–Steel–Critchlow–Fligner post hoc test was used to analyze lesion score data.

**Table 7:** Effects of tannic acid supplementation on total antioxidant capacity (TAC) in the jejunum and glutathione (GSH), oxidized GSH (GSSG), reduced GSH, and the reduced GSH/GSSG ratio in the jejunum and liver at 6 days post-infection (dpi) and 13 dpi in broiler chickens infected with *Eimeria maxima* ^1^.

		*Eimeria maxima*-Challenged ^3^						Poly. Contrasts	
Items	SCC ^2^	CC	TA0.5	TA2.75	TA5	SEM	*p*-Value	Lin.	Quad.
6 dpi jejunum									
TAC	73.67	76.98	73.62	75.93	73.12	7.27	0.72	0.54	0.79
Total GSH	42.57	31.37	30.53	24.12	20.39	10.31	0.17	0.03	0.81
GSSG	2.81	3.38	1.49	1.31	1.37	1.42	0.03	0.05	0.13
Reduced GSH ^4^	36.93	24.61	27.55	21.50	17.66	9.69	0.29	0.07	0.84
Reduced GSH/GSSG	14.67	12.77	20.03	18.32	14.61	7.98	0.32	0.86	0.20
Liver									
Total GSH	52.58	43.5	33.38	31.07	33.6	13.60	0.35	0.3	0.26
GSSG	2.35	3.13	2.36	2.42	2.06	1.47	0.59	0.28	0.82
Reduced GSH	47.87 *	37.24	28.65	26.23	29.47	11.94	0.36	0.35	0.22
Reduced GSH/GSSG	29.43 **	13.16	13.23	14.16	17.54	9	0.78	0.33	0.74
13 dpi Jejunum									
TAC	112.1	101.6	101.8	91.38	87.04	17.99	0.33	0.08	0.8
Total GSH	53.45	50.57 ^ab^	61.24 ^a^	35.07 ^b^	46.54 ^ab^	13.81	0.01	0.07	0.06
GSSG	5.45	4.70	5.20	3.57	4.10	1.30	0.13	0.10	0.22
Reduced GSH	42.54	41.18 ^ab^	50.83 ^a^	27.9 ^b^	38.33 ^ab^	12.75	0.02	0.10	0.07
Reduced GSH/GSSG	7.87	11.95	9.75	8.89	9.84	6.31	0.83	0.61	0.51
Liver									
Total GSH	40	44.15	41.37	38.95	39.3	5.36	0.27	0.11	0.30
GSSG	4 *	5.95	5.01	5.5	5.49	2.18	0.88	0.95	0.81
Reduced GSH	32	32.24	31.35	27.96	28.31	5.14	0.32	0.1	0.39
Reduced GSH/GSSG	8.56 *	5.93	6.31	5.99	5.84	1.78	0.96	0.77	0.88

^1^ SCC (sham-challenged control): broilers fed a control diet and administrated with phosphate-buffered saline via oral gavage; CC (challenged control): broilers fed a control diet and administrated with 10^4^ of *Eimeria maxima* via oral gavage; TA0.5 (tannic acid 500 mg/kg): CC + 500 mg/kg of tannic acid; TA2.75 (tannic acid 2750 mg/kg): CC + 2750 mg/kg of tannic acid; and TA5 (tannic acid 5000 mg/kg): CC + 5000 mg/kg of tannic acid. ^2^ SCC vs. CC (unpaired *t*-test): * 0.05 < *p* < 0.10, ** *p* < 0.05. ^3^ Different letters are significant different (*p* < 0.05) by PROC mixed followed by the Tukey’s multiple comparison test among *Eimeria maxima* challenged groups (CC, TA0.5, TA2.75, and TA5). Orthogonal polynomial contrasts analysis was conducted to see linear pattern (L) and quadratic pattern (Q) among *Eimeria maxima* challenged groups. ^4^ Reduced GSH = Total GSH − 2 × GSSG.

**Table 8:** Effects of tannic acid supplementation on apparent ileal digestibility of dry matter (DM), organic matter (OM), ash, and crude protein (CP) at 6 days post-infection (dpi) and 13 dpi in broilers infected with *Eimeria maxima* ^1^.

		*Eimeria maxima*-Challenged ^3^						Poly. Contrasts	
Items	SCC ^2^	CC	TA0.5	TA2.75	TA5	SEM	*p* Value	Lin.	Quad.
6 dpi									
DM	59.9	63.06	58.84	62.32	59.69	6.98	0.63	0.72	0.78
OM	65.45	65.45	61.95	65.04	63.93	6.67	0.76	0.94	0.94
Ash	12.5 **	29.18 ^a^	0.93 ^bc^	12.33 ^ab^	−22.66 ^c^	16.64	<0.01	<0.01	0.2
CP	72	71.2	64.17	69.3	67.9	5.85	0.17	0.99	0.87
13 dpi									
DM	69 **	73.85 ^b^	74.88 ^ab^	77.05 ^a^	69.67 ^c^	1.79	<0.01	<0.01	<0.01
OM	72.05 **	75.73 ^b^	77 ^ab^	78.8 ^a^	71.6 ^c^	1.77	<0.01	<0.01	<0.01
Ash	17.86 ***	47.14 ^a^	37.54 ^b^	44.87 ^a^	32.2 ^b^	4.26	4.25	<0.01	0.01
CP	68.59	73.3	68.32	69.14	68.03	5.73	0.3	0.23	0.58

^1^ SCC (sham-challenged control): broilers fed a control diet and administrated with phosphate-buffered saline via oral gavage; CC (challenged control): broilers fed a control diet and administrated with 10^4^ of *Eimeria maxima* via oral gavage; TA0.5 (tannic acid 500 mg/kg): CC + 500 mg/kg of tannic acid; TA2.75 (tannic acid 2750 mg/kg): CC + 2750 mg/kg of tannic acid; and TA5 (tannic acid 5000 mg/kg): CC + 5000 mg/kg of tannic acid. ^2^ SCC vs. CC (unpaired *t*-test): ** *p* < 0.05, *** *p* < 0.01. ^3^ Different letters are significant different (*p* < 0.05) by PROC mixed followed by the Tukey’s multiple comparison test among *Eimeria maxima* challenged groups (CC, TA0.5, TA2.75, and TA5). Orthogonal polynomial contrasts analysis was conducted to see linear pattern (L) and quadratic pattern (Q) among *Eimeria maxima* challenged groups.

**Table 9:** Effects of tannic acid supplementation on villus height (VH, μm), crypt depth (CD, μm), VH/CD, goblet cells per 100 μm VH, and goblet cells per 100 μm CD in the jejunum at 6 days post-infection (dpi) and 13 dpi in broilers infected with *Eimeria maxima* ^1^.

		*Eimeria maxima*-Challenged ^3^						Poly. Contrasts	
Items	SCC ^2^	CC	TA0.5	TA2.75	TA5	SEM	*p*-Value	Lin.	Quad.
6 dpi									
VH	1375 ***	775.6	812.26	764.79	739.04	191.88	0.91	0.56	0.9
Goblet cells per 100 μm VH	7.48	7.82	7.63	7.37	9.15	1.7	0.23	0.14	0.14
CD	248.5	279.22	357.8	354.94	328.46	55.56	0.05	0.38	0.06
Goblet cells per 100 μm CD	4.05	3.94	3.74	3.62	3.97	0.49	0.5	0.83	0.14
VH/CD	5.8 ***	2.87	2.49	2.22	2.39	0.82	0.51	0.31	0.3
13 dpi									
VH	1344 **	973.9	1101.3	1210.8	1299.3	245.9	0.1	0.02	0.59
Goblet cells per 100 μm VH	5.12 ***	8.53	10.12	8.35	8.67	1.75	0.24	0.42	0.83
CD	248.62 **	310.36	289.7	315.88	329.65	42.68	0.21	0.11	0.74
Goblet cells per 100 μm CD	2.72 ***	4.46	4.34	4.14	3.87	0.96	0.69	0.24	0.99
VH/CD	5.81 ***	3.22	4.09	3.95	4.07	0.85	0.20	0.21	0.45

^1^ SCC (sham-challenged control): broilers fed a control diet and administrated with phosphate buffered saline via oral gavage; CC (challenged control): broilers fed a control diet and administrated with 10^4^ of *Eimeria maxima* via oral gavage; TA0.5 (tannic acid 500 mg/kg): CC + 500 mg/kg of tannic acid; TA2.75 (tannic acid 2750 mg/kg): CC + 2750 mg/kg of tannic acid; and TA5 (tannic acid 5000 mg/kg): CC + 5000 mg/kg of tannic acid. ^2^ SCC vs. CC (unpaired *t*-test): ** *p* < 0.05, *** *p* < 0.01. ^3^ *Eimeria maxima*-challenged groups (CC, TA0.5, TA2.75, and TA5) were compared by PROC MIXED followed by the Tukey’s multiple comparison test. Orthogonal polynomial contrasts analysis was conducted to see linear pattern (L) and quadratic pattern (Q) among *Eimeria maxima* challenged groups.

## Data Availability

The data presented in this study are available on request from the corresponding author.
